# Comparative Exposure Assessment of ESBL-Producing *Escherichia coli* through Meat Consumption

**DOI:** 10.1371/journal.pone.0169589

**Published:** 2017-01-05

**Authors:** Eric G. Evers, Annemarie Pielaat, Joost H. Smid, Engeline van Duijkeren, Francy B. C. Vennemann, Lucas M. Wijnands, Jurgen E. Chardon

**Affiliations:** 1 Centre for Infectious Disease Control (CIb), National Institute for Public Health and the Environment (RIVM), Bilthoven, The Netherlands; 2 Institute for Risk Assessment Sciences (IRAS), Utrecht University, Utrecht, The Netherlands; 3 Public Health and Health Services Division, National Institute for Public Health and the Environment (RIVM), Bilthoven, The Netherlands; Universiti Putra Malaysia, MALAYSIA

## Abstract

The presence of extended-spectrum β-lactamase (ESBL) and plasmidic AmpC (pAmpC) producing *Escherichia coli* (EEC) in food animals, especially broilers, has become a major public health concern. The aim of the present study was to quantify the EEC exposure of humans in The Netherlands through the consumption of meat from different food animals. Calculations were done with a simplified Quantitative Microbiological Risk Assessment (QMRA) model. The model took the effect of pre-retail processing, storage at the consumers home and preparation in the kitchen (cross-contamination and heating) on EEC numbers on/in the raw meat products into account. The contribution of beef products (78%) to the total EEC exposure of the Dutch population through the consumption of meat was much higher than for chicken (18%), pork (4.5%), veal (0.1%) and lamb (0%). After slaughter, chicken meat accounted for 97% of total EEC load on meat, but chicken meat experienced a relatively large effect of heating during food preparation. Exposure via consumption of filet americain (a minced beef product consumed raw) was predicted to be highest (61% of total EEC exposure), followed by chicken fillet (13%). It was estimated that only 18% of EEC exposure occurred via cross-contamination during preparation in the kitchen, which was the only route by which EEC survived for surface-contaminated products. Sensitivity analysis showed that model output is not sensitive for most parameters. However, EEC concentration on meat other than chicken meat was an important data gap. In conclusion, the model assessed that consumption of beef products led to a higher exposure to EEC than chicken products, although the prevalence of EEC on raw chicken meat was much higher than on beef. The (relative) risk of this exposure for public health is yet unknown given the lack of a modelling framework and of exposure studies for other potential transmission routes.

## 1. Introduction

During the last decade, EEC (= extended-spectrum β-lactamase (ESBL) and plasmidic AmpC (pAmpC) producing *Escherichia coli*) from food animals, especially from broilers, have become a major public health concern because of the possible transmission of these bacteria or their plasmid-encoded resistance genes to humans [1]. It has been suggested that EEC strains from animal origin can cause human infections. Infections with resistant bacteria are associated with higher rates of illness and death [2]. EEC can play a role in a variety of infections, including community-acquired urinary tract infections [3]. Exposure of humans to EEC or their resistance genes occurs via the food chain, by direct contact or via the environment [1, 2, 4]. Direct contact with broilers has been identified as a risk factor for carriage of EEC for humans on broiler farms [1]. The prevalence of EEC among Dutch broilers is high and broilers might therefore form a reservoir from where spread of these resistant bacteria to humans in the general population might occur [1, 5, 6]. Similar ESBL-genes and plasmids carrying these genes have been found in broilers, chicken meat and clinical isolates from humans. Transmission of EEC from broilers to humans through the food chain has therefore been proposed [2, 7–9]. These bacteria, however, have also been identified in other food animals such as veal calves, dairy cattle and pigs and on meat and meat products originating from these animals, although with much lower prevalence [10]. As the prevalence of EEC was highest in broilers and poultry meat, it has been suggested that poultry meat is the major contributor to human exposure through food [7, 8]. Currently, much effort is directed towards determining the contribution of various livestock reservoirs to human colonization and infection with ESBL/pAmpC-producing bacteria, especially *E*. *coli*. In The Netherlands the prevalence of carriage of ESBL/pAmpC- producing Enterobacteriaceae in the general population was 5–10% [11–13]. To date, the relative importance of the various transmission routes is unknown. In order to design effective intervention strategies to reduce the exposure of humans to EEC, quantitative data on human exposure through the various transmission routes are needed. Most investigations, however, only reported presence/absence of EEC and not the concentrations of these bacteria [4] and none quantify human exposure.

The objective of the present study was to quantify human exposure to EEC through the consumption of meat from different food animals and through specific meat products, which was done using a Quantitative Microbiological Risk Assessment (QMRA) model.

## 2. The Model

### 2.1. Model description

**Fig 1:**
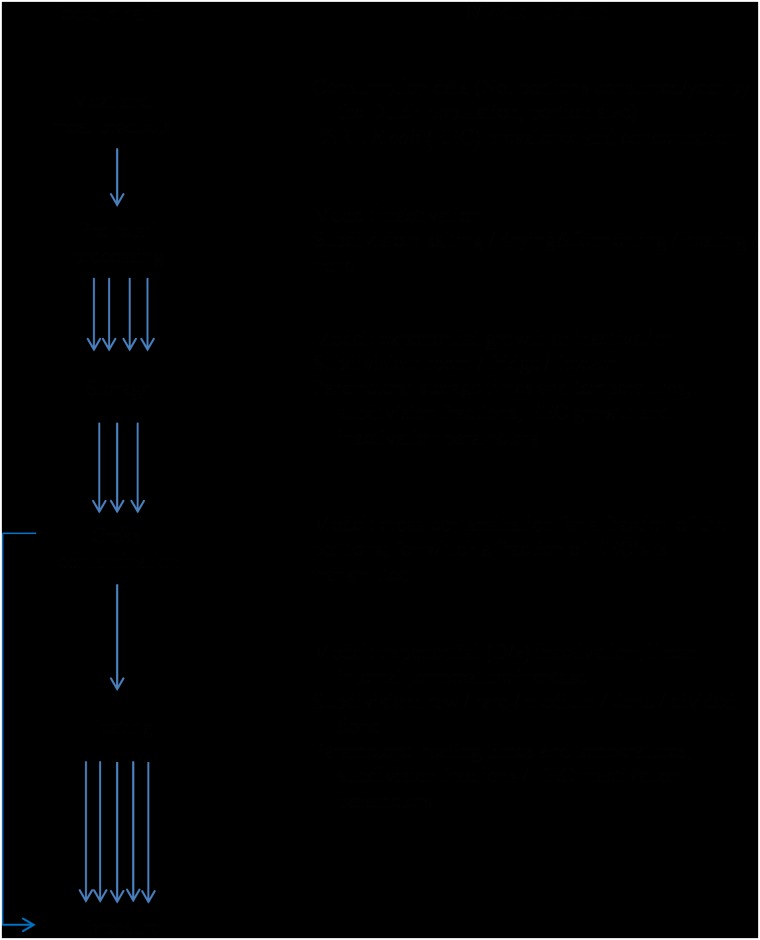
Overview of the sQMRA model used for the calculations. In- and output (text box with straight corners), processes (rounded corners) and details (dashed lines) are shown.

The model (Fig 1) used is based on the swift Quantitative Microbiological Risk Assessment (sQMRA) approach [14], which is a simplified QMRA calculation. The exposure part of that model describes the change in the number of a microorganism on a food product, starting at retail and incorporating the effect of preparation (cross-contamination, heating) at the consumers' home. The present model extends this with pre-retail processing, storage at the consumers’ home and more comprehensive modelling of the effect of heating. Also, calculations are executed simultaneously for all meat and meat products, where first calculations are performed on the level of contaminated products and then at the population level (i.e. exposure over one year for the Dutch population). The model as applied for the transmission of EEC is described below, focusing on extensions from [14].

Pre-retail processing such as smoking, salting, drying and cooking is accounted for by including a reduction factor <1 with which raw meat concentrations are multiplied to obtain realistic retail concentrations.

Storage at the consumer’s home is distinguished in three categories: at room temperature, in the refrigerator and in the freezer. Growth of microorganisms, which occurs above the minimum growth temperature, is described by an exponential primary growth model combined with the temperature-dependent part of the gamma model as secondary growth model [15]:

$$G={\text{e}}^{\left(\frac{\text{ln2}}{t_{gen}}\right)\cdot t_{st}}$$ (1)

$$t_{gen}=\frac{t_{gen\_min}}{{\left(\frac{T_{st}-T_{min}}{T_{opt}-T_{min}}\right)}^{2}}$$ (2)

Where *G* is the EEC growth multiplication factor, *t*_*gen*_ is the EEC generation time, *t*_*st*_ is the storage time, *t*_*gen_min*_ is the EEC minimum generation time, *T*_*st*_ is the storage temperature, *T*_*min*_ is the EEC minimum growth temperature and *T*_*opt*_ is the EEC optimum growth temperature.

EEC is limited to a maximum population density. Inactivation is described as an exponential decrease with storage time for room and refrigerator and with a time-independent factor for the freezer.

Cross-contamination during preparation by the consumer is described as a process in which a fraction of the microorganisms on the meat product is transmitted to the hands of the cook or kitchen utensils (cutting board, knife, etc.) prior to heating and an assumed identical fraction is subsequently transmitted from the hands or kitchen utensils to raw vegetables when prepared by the consumer together with the meat product.

Three types of food products are distinguished based on different characteristics in relation to inactivation of microorganisms during the heating process in the kitchen and which are therefore modelled differently:

- S (surface), where the microbial contamination is only on the meat surface. These are whole pieces of meat, from large (steak) to small (strips), but also bacon and some ham types.
- I (internal), where the microbial contamination is mainly on the inside of the product. This includes roulades, cordon blue, minced meat products (e.g. roll, oliver, steak tartare, sausages, burgers) and products that are pasted and/or squeezed (schnitzels, nuggets, luncheon meats). Surface contamination is neglected in the calculations.
- D (dividable), which is minced meat. This is distinguished as a separate category as it can be heated either as a whole (meatball; microbial contamination on the inside so heating effect like category I), or divided into crumbs, i.e. very small pieces (microbial contamination (almost) at the surface so that heating effect can be estimated as for category S).

The D/z-inactivation model [16] is used, which is applied in a different way for each of the three types of food products described above (S, I, D) because of the location of the microorganisms in/on the product. Further the heating categories raw, rare, medium, done and (specifically for D-products) divided-done are distinguished. Heating is effective on the fraction of bacteria that is not cross-contaminated.

For S-products,

$$R=\frac{t_{he}\cdot 10^{\left(\frac{T_{end}-T_{ref}}{z}\right)}}{D_{ref}}$$ (3)

$$p_{he}={\text{10}}^{-\;R}$$ (4)

where *R* is the log_10_ reduction of the number of EEC, *p*_*he*_ is the fraction of EEC that survive heating, *t*_*he*_ is the product-specific heating time, *T*_*end*_ is the product-specific final heating temperature (which is reached immediately for type S), *T*_*ref*_ is the reference temperature at which *D*_*ref*_ was estimated, *z* is the temperature increase needed to reduce the *D* value with a factor 10 and *D*_*ref*_ is the decimal reduction time at temperature *T*_*ref*_.

For I-products, the model of Møller *et al*. [17] is applied, which assumes that microorganisms only survive in the core of the product. A linear increase of temperature in the core from start temperature *T*_*0*_ to end temperature *T*_*end*_ is assumed. *T*_*0*_ will be equal to the storage temperature for room and fridge; when the product is stored in the freezer *T*_*0*_ will usually be fridge temperature (assuming the consumer thaws the product prior to heating), except for snacks where *T*_*0*_ is freezer temperature. The log_10_ reduction *R* is estimated numerically, dividing the temperature trajectory into 10 segments:

$$R=\frac{t_{he}}{10D_{ref}}\sum_{i=0}^{9}10^{\frac{T_{0}+\left(0.05+\frac{i}{10}\right)\left(T_{end}-T_{0}\right)-T_{ref}}{z}}$$ (5)

and

$$p_{he}=C{\text{10}}^{-\;R}$$ (6)

where the size of the core fraction *C* is assumed to be a third of the portion size [17].

For D-products, eqs (5) and (6) are used when the product is heated as a whole (categories raw, rare, medium and done) and eqs (3) and (4) when the product is divided (category divided-done). Parameters values for *t*_*he*_ and *T*_*end*_ can differ for done and divided-done.

The number of microorganisms ingested per contaminated portion of meat (product) equals the sum of cross-contaminated microorganisms and microorganisms that survive heating. Finally, this results in 24 categories for S- and I-food products: 3 storage (room/fridge/freezer) x 2 cross contamination (yes/no) x 4 heating (raw/rare/medium/done) categories, and 30 categories for D-food products (which have 5 heating categories: raw/rare/medium/done/divided-done).

As a last step, the output at population level (consumption by the whole Dutch population in a year) is calculated. This consists of the number of contaminated portions at the moment of consumption and the total number of microorganisms ingested, obtained by combining:

- the calculated number of microorganisms ingested per contaminated portion for each of 24 or 30 categories per food product;
- the number of consumed portions in a year by the Dutch population;
- the subdivision fractions for each of the storage, cross-contamination and heating categories;
- EEC prevalence (fraction of contaminated portions) at (pre-)retail and at the moment of consumption. The prevalence at the moment of consumption is obtained by multiplying the prevalence at (pre-)retail by 1 minus the Poisson probability of 0 CFU, given the number of CFU in a portion at the moment of consumption (CFU = colony forming unit) and assuming randomly distributed EEC.

### 2.2. Parameter values

The parameter values were based on data from the literature or were estimates from the authors as experts in the field of food safety, food consumption and microbiology. Table 1 gives an overview of information sources and their characteristics that were used to estimate model parameter values.

**Table 1:** Overview of information sources.

Section	Model parameters	Main information sources	Information source characteristics
2.2.1	EEC prevalences of raw meat	[10]	Annual surveillance of retail samples in the Netherlands; 2909 meat (product) samples
2.2.2	EEC raw meat concentrations	[18–20]	EEC Measurements on 200 retail chicken fillets in The Netherlands. *E*. *coli* measurements from beef, pork and chicken meat (2875 samples)
2.2.3	Preprocessing treatments	[21]	Dutch butchers manual. 11 meat product categories
	Reduction of EEC through heating	[22, 23]	Overview reports by organizations of experts
	Reduction of EEC in dry sausages	[24]	Literature review on fermentation, drying and storage
	Reduction of EEC through salting	[25–27]	Experimental results
2.2.4	Consumption of meat (frequency, quantity) and raw vegetables	[28–30]	Three large Dutch National Food Consumption Surveys; data on food consumption in The Netherlands from 5837 persons during two days, distinguishing 246 meat products
2.2.5	Storage time and temperature profiles	[31]	Food consumption and food handling survey in The Netherlands; 2226 respondents. Four meat product storage profiles
	EEC growth and reduction properties	[27, 32, 33]	Experimental results
2.2.6	Cross-contamination rate of EEC	This study	Literature study performed for this paper, using 30 papers
2.2.7	Food heating profiles	[29, 31, 34, 35]	Consumer surveys in The Netherlands complemented with expert estimates. 16 meat product heating profiles
	Heating times and temperatures	[36, 37]	Dutch cookbook and advised temperatures from practice
	EEC reduction properties	[16]	Literature review on thermal inactivation of food pathogens

#### 2.2.1 EEC prevalences of raw meat

EEC prevalences in The Netherlands determined for the annual surveillance of antimicrobial resistance as reported in Table ESBL04 in MARAN [10] report were used. In 2014, in total 2909 meat/meat product samples were analysed for EEC using selective enrichment and selective culturing. Data of 2826 meat/meat products were included in the present analysis: 917 originating from cattle, 29 from calves, 1306 from pigs, 526 from chickens and 48 from lamb. Data on meat samples from turkeys (n = 44) and imported chicken meat (n = 39) were excluded in the present study. The EEC prevalences were (first number is for fresh meat, second number is for meat product) 2.2% and 7.8% for beef, 3.1% and 21.0% for veal, 2.7% and 4.0% for pork and 0% and 0% for mutton. For chicken, fresh meat prevalence was 67.0% and this same number was used for meat products as for chicken meat products no data were available. All meat (products) from the Dutch National Food Consumption Survey were assigned to ‘fresh meat’ or ‘meat product’ according to the categorization used by The Netherlands Food and Consumer Product Safety Authority.

#### 2.2.2 EEC raw meat concentration

The only published concentration data on EEC were measured on retail chicken fillets in The Netherlands and the value for contaminated conventional chicken fillet was 8.57 CFU/g [18, 19]. For meat from other food animals no EEC concentration data were available. *E*. *coli* concentration data on beef, pork and poultry meats in Belgium [20] were used to estimate EEC concentration on contaminated non-chicken meat, thus assuming that the EEC/*E*.*coli* ratio was a constant. The 95th percentile *E*. *coli* values on cuts and minced meat (beef and pork) and fillet and prepared meat (for chicken) from 2002 and 2003 were used. Compared to chicken, the *E*. *coli* values for beef and pork were 0.991 and 0.809 log_10_ lower, respectively. The EEC concentrations for beef and veal and for pork were subsequently estimated at 0.875 and 1.33 CFU/g, respectively.

#### 2.2.3 Pre-retail EEC reduction

Part of the large variety of meat products for sale in The Netherlands are processed before being placed in retail shops and these can be categorized according to the pre-retail processing method applied into (sub)groups 1–8 (Table 2). The preprocessing steps for each of these categories were based on a guide for the production of meat products [21]. An additional group (group 9) includes products that were heated before retail and reheated before consumption. The characteristics of pathogenic *E*. *coli* in terms of temperature, pH and water activity growth range and heat, salt, drying and freezing resistance can be found in Wareing *et al*. [38]. Assuming that EEC behaves similar to pathogenic *E*.*coli*, heating was the process that gives by far the largest reduction of EEC numbers. The estimate of the effect of heating was based on two reports. ILSI [22] gave an overview of so-called safe harbours, which are ‘generally recognized processes and process criteria that have been established over time by consensus or by regulation’ and applicable to bacteria in general. The ILSI [22] overview included a 7, 6.5 and 6 log_10_ reduction for *Salmonella* in Ready-to-Eat (RTE) poultry products, *Salmonella* in RTE beef products and non-proteolytic *Clostridium botulinum* in cooked chilled foods, respectively. ACMSF [23] advised to heat for 2 min at 70°C to give at least a 6 log_10_ reduction of *E*. *coli* O157:H7, *Salmonella* spp. and *Listeria monocytogenes* in meat products. Based on the above, a 6.5 log_10_ reduction in case of pre-retail heating of meat was used (Table 2).

**Table 2:** Pre-retail categories.

Meat product categories			Processing characteristics	EEC reduction (log_10_) used for calculations
No.	Description	Example product		
1	blood sausage types	blood sausage	cooking/heating of ingredients, final treatment heating at 80–85°C, 15 min per cm diameter	6.5
2	fried products	cold cuts of roast beef	frying at 175°C—15 min/cm height or heating at 75°C to core-temperature of 65°C and frying	6.5
3	bologna	cooked sausage (e.g. Mortadella style)	drying at 40°C and 80% relative humidity, smoking at 40°C and heating at 75–78°C to core temperature of 70°C	6.5
4	liver sausage types	liverwurst	heating at 75–80°C, 15 min/cm diameter	6.5
5	dry sausage types	salami	ripening by drying and fermentation with lactic acid bacteria, lowering of water activity to ≤ 0.90	1.3
6a	specialities with germ reduction	liver paste	various treatments	6.5
6b	specialities without germ reduction	filet americain^a^	no treatment (like raw ground beef)	0
7a	salted products—heated	ham	salting, smoking and heating to core temperature of 70°C or 1 hour per kg product	6.5
7b	salted products—dried	smoked beef	salting, smoking and drying to weight loss of approximately 15%	2.1
8	canned products	corned beef	final treatment: canning and autoclaving (12-log_10_ reduction principle)	12
9	products to be reheated	chicken nuggets	pasteurization (various temperatures)	6.5

Subdivision of pre-retail processed meat products in terms of processing characteristics and the size of EEC reduction used in the model calculations. These products are mainly consumed on sandwich (category 9 excepted). For explanation see 2.2.3.

^a^ filet americain is a spread of raw minced beef mixed with a spicy sauce.

For dry sausages (category 5), the effect of fermentation and drying in the production stage was estimated at an average reduction of 1.3 log_10_ using the overview of Hwang *et al*. [24] and Glass *et al*. [25], Heir *et al*. [39] and Ducic *et al*. [40]. Storage gave an additional reduction (see 2.2.5).

To estimate the effect of salting (category 7b), a loglinear model was fitted to inactivation data retrieved from Glass *et al*. [25], Ryu *et al*. [26] and ICMSF [27]. Average salt percentage and salting time were derived from [21] to 18% and 3.5 days, respectively. Average inactivation was then 2.1 log_10_.

#### 2.2.4 Food consumption

The consumption of meat (frequency, quantity) and the consumption of meat eaten together with raw vegetables (frequency) was estimated for the general population in The Netherlands: data from three large Dutch National Food Consumption Surveys, among children aged 2 to 6 years old [28], children and adults aged 7 to 69 years old [29], and community dwelling older adults aged 70 years and older [30] were used for the analyses. Food intake data were collected by two diaries and/or two 24-hr dietary recalls (method differed by age category) and entered into the EPIC-Soft ^®^ computer program [41]. Using this approach it was possible to assess the food consumption in detail by hour and place. More information on the three dietary surveys can be found on the website [42].

For each survey, both data on all consumed meat products and on the same meat products eaten together with raw vegetables were identified. A time frame of one hour was used to define whether a meat product was eaten together with raw vegetables. Subsequently, for each meat product, 1) the total number of eating occasions, 2) the mean meat quantity consumed during these occasions (excluding bone weight), and 3) the total number of eating occasions together with raw vegetables, was calculated. The quotient of 3) and 1) was used as an estimate for the fraction of portions with potential cross-contamination. The results were weighted to correct for small deviances between the survey population and the Dutch population in socio-demographic characteristics and for day of the week and season of data collection. The total consumption data for The Netherlands was obtained by combining the results from the three dietary surveys, weighing the results for the size of the three study populations and of the corresponding age groups in the Dutch population.

Part of the meat food products in the Dutch National Food Consumption Survey were not directly assigned to a food animal (beef, veal, pork, etc.): the categories ‘fresh meat—unclassified’ and ‘processed meat’. In case the food animal was not clear from the product description, a guide for the production of meat products [21] and internet [43] was used to assign these products to food animals. For a number of terms that occurred in multiple product names assumptions were as follows: minced meat, sausage and roulade (all unspecified) and ham, bacon and shoarma were assigned to pork; hamburger was assigned to beef; ‘meat unspecified’ was for 50% assigned to pork and 50% to beef, liver products contained 25% pork.

For calculations at food animal level, in case a food product from the Food Consumption Survey could originate from meat of different food animals (10 products; 0.9% of the total number of portions), the number of portions consumed as reported by the survey was divided among these food animals according to estimated fractions for these animals, retaining portion size. In case a food product was a mixture of meat originating from two different food animals (5 products; 10% of the total number of portions), the original number of portions as reported by the survey was allocated to both food animals, but the reported portion size was reduced for the respective food animals according to estimated fractions for these animals.

For calculations at food product level for the products described above, portion numbers or sizes from different food animals were combined again. In the final dataset 62 beef, 14 veal, 143 pork, 16 mutton/lamb and 27 chicken products were considered, in total 246 meat products (less than the sum due to the multi food animal products).

Mean, 2.5 and 97.5 percentile values were 75.0, 15.0 and 186.4 g for portion size and 0.47, 0.00 and 1.00 for fraction of portions with potential cross-contamination. Numbers of portions consumed per year in The Netherlands are given in the Results section.

#### 2.2.5 EEC growth or reduction during storage at the consumer’s home

**EEC characteristics** The following data sources were consulted for references to scientific literature: Google, Scopus, Combase, published QMRA’s and ICMSF [27]. Identified original research papers and meta-analysis papers were studied. For every parameter, extensive discussion among the authors led to selection of the most appropriate data source and interpretation of the data, resulting in the parameter values in Table 3. For dry sausages (pre-processing category 5) a temperature specific reduction during storage at the consumer (due to low pH and a_w_) was estimated using the overview of Hwang *et al*. [24]. Fraction of survival per day was 0.773 and 0.902 at room and fridge temperature, respectively (corresponding to 0.112 and 0.045 log_10_ reduction per day, respectively). It was assumed that for pre-processing category 8 (canned products; Table 2) EEC numbers were constant during storage.

**Table 3:** Values used for EEC growth and survival parameters related to storage.

Parameter description	Parameter value	Source
Minimum generation time (h)	0.47	[27] p.132 and [32]
Optimum growth temperature (°C)	37.5	[27] p.131
Minimum growth temperature (°C)	6	[32]
Max. population density (CFU/g)	1.23E+05	[33]
Fraction survival room (/day)	1	[27] p. 131
Fraction survival fridge (/day)	1	[32]
Fraction survival freezer	0.1	[27] p. 129

The values were based on data for non-pathogenic and pathogenic *E*.*coli* including *E*. *coli* O157.

**Storage characteristics** Based on Chardon and Swart [31], four storage profiles were identified (Table 4). Storage time at room temperature was set at 1/5^th^ of the storage time in the fridge, except for shelf-stable meat products (7 d). Freezer survival was modelled as time independent. Room and fridge temperature were set at 18°C (authors estimate) and 5.99°C [44], respectively.

**Table 4:** Storage profiles for meat products at the consumers home.

Profile		Fraction of portions stored in:			Storage time (d) in:	
No.	Description	Room	Fridge	Freezer	Room	Fridge
1	meat to be cooked	0.0067	0.43	0.56	0.42	2.1
2	shelf-stable meat products	0.19	0.80	0.010	7.0	6.2
3	meat products to be consumed raw	0.0019	0.97	0.030	0.52	2.6
4	perishable meat products	0.010	0.97	0.020	0.86	4.3

#### 2.2.6 Cross-contamination of EEC through meat preparation by the consumer

As stated in the model description (section 2.1), cross-contamination was considered as the transmission process of EEC from the meat product to raw vegetables which were prepared at the same time. The model of Gkogka *et al*. [45] was used, extending it with the route via washed hands (Table 5). Transmission via cutting boards and utensils was considered negligible when washed with soap, or replaced by clean ones. A literature study (2000-present) was performed to obtain the parameter values [45–74]. An estimated fraction of 1.5E-3 of the microorganisms was transmitted (Table 5). Please note that to obtain the fraction of microorganisms transmitted for a specific meat product, this number must be multiplied with the fraction of portions of this specific meat product that was prepared together with raw vegetables. The latter fractions were obtained from the food consumption survey data (see 2.2.4). Finally, it was assumed that the value of 1.5E-3 was applicable for all products and product categories (S, I, D). However, products which were not heated (heating category 12; see 2.2.7) and not sliced by the consumer were assumed to give no cross-contamination (fraction transmitted = 0).

**Table 5:** Calculation of the extent of cross-contamination.

Transmission route considered	Probability of occurrence of transmission route (P)	Fraction of microorganisms transferred to raw vegetables via transmission route (F)	P x F
Unwashed cutting board and utensils	3.4E-2	2.2E-2	7.4E-4
Rinsed cutting board and utensils	0.28	3.2E-3	9.0E-4
Unwashed hands	0.12	4.5E-3	5.2E-4
Washed hands	0.88	6.1E-5	5.4E-5
Quantity		calculation	
Overall transmission		Sum of P x F	2.2E-3
Probability of preparing raw vegetables after meat product (Q)			0.68
Fraction of microorganisms transmitted		Q x (Sum of P x F)	1.5E-3

Cross-contamination occurs during preparation of a meal consisting of a meat product and raw vegetables. For explanation see 2.2.6. For example, in 88% of the cases the food preparer washed his/her hands and then 0.0061% of the microorganisms on the meat ends up in the raw vegetables via his/her hands.

#### 2.2.7 EEC reduction through heating of meat by the consumer

**EEC characteristics** Values from van Asselt and Zwietering [16] for *E*. *coli* were used. These were *T*_*ref*_ = 70°C, z = 10.6°C and log_10_ *D*_*ref*_ = -0.67 log_10_ min.

**Heating characteristics** Sixteen preparation profiles for meat in The Netherlands were set by the authors (Table 6). Data on preferred preparation styles were obtained for minced meat and minced meat preparations [29, 31], hamburger [34], beefsteak and steak tartare [31] and pan fried sausage [35]. For fresh beef, veal, lamb and pork in general, two types were distinguished: intact cuts of meat of approximately one serving size and large chunks of meat of approximately four serving sizes. For fresh meat in general, no data on preparation were available so the following assumptions were made: beef, veal and lamb were prepared rare, medium or done; pork was prepared medium or done and chicken was always prepared done. The remaining meat products were described with the profiles meat strips, unspecified, reheated sausage and braised meat. The exact values for the fractions of portions prepared were in a next step set by the authors after extensive discussion.

**Table 6:** Heating profiles for food products at the consumers’ home.

Profile		Fraction of portions					Preparation parameters for a selected style		
No.	Description	raw	rare	medium	done	divided-done	style	core *T*_*end*_ (°C)	*t*_*he*_ (min)
1	Minced meat	0	0	0.023	0.543	0.434	done	71.5	20^a^
2	Hamburger	0	0.025	0.111	0.864	0	medium	61.5	5
3	Beefsteak	0	0.117	0.738	0.145	0	medium	61.5	5
4	Steak tartare	0.095	0	0.514	0.390	0	medium	61.5	5
5	Pan fried sausage	0	0	0.139	0.861	0	done	71.5	20
6	Beef/veal/ lamb large	0	0.10	0.65	0.25	0	medium	61.5	25
7	Beef/veal/ lamb cut	0	0	0.25	0.75	0	medium	61.5	5
8	Pork large	0	0	0.75	0.25	0	medium	61.5	25
9	Pork cut	0	0	0	1.00	0	done	71.5	10
10	Chicken	0	0	0	1.00	0	done	71.5	15
11	Meat strips	0	0	0	1.00	0	done	90	7
12	RTE meat	1.00	0	0	0	0	raw	18	0
13	Unspecified	0	0	0	1.00	0	done	71.5	15
14	Reheated sausage	0	0	0	1.00	0	done	80	8
15	Minced meat preparation	0	0	0.04	0.96	0	medium	61.5	5
16	Braised meat	0	0	0	1.00	0	done	90	120

For explanation see 2.2.7.

^a^: 6 min for divided minced meat. RTE = ready to eat.

Preparation styles were expressed in terms of a core temperature *T*_*end*_ and a heating time *t*_*he*_ (Table 6). For I- and D-undivided products (Eq 5) in general, 52, 61.5 and 71.5°C was set for rare, medium and done core end temperatures, respectively [36]. Core end temperature for meat strips was set at 90°C [75]. Core end temperatures for reheated sausage and braised meat were estimated by the authors. For S- and D-divided products, temperature was set at 90°C (based on Lahou *et al*. [75]) for the entire heating time (Eq 3).

Heating times *t*_*he*_ were based on Henderson *et al*. [37] except for meat strips [75]. Heating times *t*_*he*_ for preparation styles not given in Table 6 were calculated using the ratio 0.8–1.0–1.2 for rare—medium—done, which followed from the accessory *T*_*end*_ values and the assumed linear temperature increase in the core [17] starting from fridge temperature.

Core fraction and T_0_-values were explained in 2.1 (Model description). It was assumed that EEC numbers do not change in the consumer phase for raw (unheated) products.

### 2.3. Sensitivity analysis

A sensitivity analysis was performed on a selection of parameters from different phases that were considered to be most uncertain and have a large effect on model output, based on expert judgement. These parameter values were divided or multiplied by 10 for the sensitivity analysis.

## 3. Results

Aggregating on food animals (Table 7, first column) and considering the portion level at the moment of consumption, the exposure per portion (Table 7, fourth column) was highest for beef and chicken. This exposure was the mathematical product of the exposure per contaminated portion (highest for beef) and the fraction of contaminated portions (highest for chicken). Meat preparation by the consumer lowered EEC numbers per portion so that the fractions of contaminated portions as shown in the third column in Table 7 (calculated by Poisson variability; see section 2.1) were lower than the EEC raw meat prevalences (section 2.2.1). Considering the population level (consumption by the Dutch population in a year) and the moment of consumption, the human exposure to EEC through a meal containing meat was highest through beef, which accounted for 78% of total exposure, and through chicken (18%). Pork and veal were less important and mutton/lamb gave no EEC exposure (Table 7, last column). This exposure was the mathematical product of the exposure per portion and the total number of consumed portions. The total number of consumed portions was highest for pork and lowest for veal.

**Table 7:** Contribution of food animals to the exposure of humans to EEC through meat at the moment of consumption.

Category	exposure per contaminated portion (No. EEC/portion)	fraction of contaminated portions	exposure per portion (No. EEC/portion)	total number of consumed portions	total exposure (No. EEC)
Beef	1.88E+1	1.46E-2	2.75E-1 (1)	3.29E+9	9.05E+8 [77.5%]
Chicken	1.75E+0	6.85E-2	1.20E-1 (2)	1.75E+9	2.09E+8 [17.9%]
Pork	2.44E+0	3.05E-3	7.44E-3 (4)	7.12E+9	5.29E+7 [4.5%]
Veal	3.56E+0	1.35E-2	4.81E-2 (3)	2.81E+7	1.35E+6 [0.1%]
Mutton/lamb	N.a.	0.00E+0	0.00E+0 (5)	5.22E+7	0.00E+0 [0%]
Mean (^m^) or sum (^s^)	6.15E+0^m^	1.55E-2^m^	9.55E-2^m^	1.22E+10^s^	1.17E+9^s^

Results are sorted for total exposure, ranked from high to low. The total number of consumed portions and exposure refer to the consumption by the Dutch population in a year. No. = number, N.a. = not available. (): rank high to low. []: total exposure per animal as percentage of overall total exposure.

Considering separate food products at the portion level at the moment of consumption, the exposure to EEC per portion was highest for teewurst, ossenworst and filet americain (Table 8, fifth column; see caption for a description of these products). The full top 10 of EEC exposure per portion (fifth column) consisted on the one hand of chicken products which were characterized by a low dose per contaminated portion (third column) and a high fraction of contaminated portions (fourth column), and on the other hand of non-chicken products for which it was the other way around.

**Table 8:** Contribution of different food products to the exposure of humans to EEC through meat at the moment of consumption.

food animal	product	exposure per contaminated portion (No. EEC/portion)	fraction of contami-nated portions	exposure per portion (No. EEC/portion)	total number of consumed portions per year	total exposure (No. EEC per year)
Beef	Filet americain^a^	2.86E+1	7.79E-2	2.23E+0 (3)	3.21E+8	7.16E+8 [61.3%]
Chicken	Chicken fillet	1.73E+0	1.25E-1	2.17E-1 (11)	6.77E+8	1.47E+8 [12.6%]
Beef	Ossenworst^b^	2.92E+1	7.79E-2	2.27E+0 (2)	4.46E+7	1.01E+8 [8.7%]
Beef	Steak tartare	1.26E+1	4.25E-2	5.35E-1 (5)	8.76E+7	4.69E+7 [4.0%]
Beef	Hamburger	6.06E+0	1.34E-2	8.13E-2 (21)	2.80E+8	2.28E+7 [2.0%]
Pork	Teewurst^c^	6.16E+1	4.00E-2	2.46E+0 (1)	6.44E+6	1.59E+7 [1.4%]
Beef&Pork	Salami	3.20E+0	1.72E-2	5.51E-2 (31)	2.34E+8	1.29E+7 [1.1%]
Beef&Pork	Cervelat	2.46E+0	1.94E-2	4.77E-2 (33)	2.66E+8	1.27E+7 [1.1%]
Chicken	Chicken leg	2.00E+0	1.27E-1	2.55E-1 (10)	4.61E+7	1.18E+7 [1.0%]
Chicken	Chicken drumstick	1.58E+0	1.21E-1	1.91E-1 (14)	5.51E+7	1.06E+7 [0.9%]
Chicken	Chicken roast	2.15E+0	2.03E-1	4.38E-1 (7)	2.29E+7	1.00E+7 [0.9%]
Chicken	Chicken half	2.59E+0	2.32E-1	6.02E-1 (4)	9.08E+6	5.46E+6 [0.5%]
Chicken	Chicken strips	1.65E+0	2.14E-1	3.55E-1 (8)	8.25E+6	2.92E+6 [0.3%]
Chicken	Chicken snacks	1.96E+0	1.40E-1	2.74E-1 (9)	5.99E+6	1.64E+6 [0.1%]
Veal	Veal roulade	5.10E+0	8.64E-2	4.41E-1 (6)	7.10E+5	3.13E+5 [0.0%]

The table upper part shows the 10 products with highest total exposure, ranked from high to low. This was supplemented with products that complete the top 10 of products with highest exposure per portion (lower part of the table). The total number of consumed portions and total exposure refer to the consumption by the Dutch population in a year. (): rank number high to low on the list of 246 products. []:total exposure per product as percentage of overall total exposure.

^a^filet americain = spread of raw minced beef mixed with a spicy sauce,

^b^ossenworst = sausage of raw minced beef with salt and spices added,

^c^Teewurst = sausage of spreadable raw minced pork and bacon with salt and spices added.

Considering the properties of the top 10 products with the highest exposure per portion (Table 8, fifth column), the top three of the ranking (Teewurst, Ossenworst, Filet americain) all belonged to pre-retail processing category 6b: products that are unprocessed during pre-retail and consumed raw (Table 2). For these products, EEC numbers were not reduced in pre-retail nor during the consumer phase (no heating).

The top 10 further included five chicken products (ranked 4, 7, 8, 9, 10) which were characterized by high EEC prevalence and concentration, no EEC number reduction in pre-retail and S-products (section 2.1) heated done so that all EEC were transmitted via the cross-contamination route (see previous paragraph). The differences in exposure per portion for these chicken products were related to differences in portion size and the fraction of portions with potential cross-contamination (i.e., preparation together with raw vegetables).

The sole representative of heating profile 4 (Table 6) was steak tartare which was ranked No. 5. Next to the pre-retail processing category 6b products, this was the only product that combined no EEC number reduction in pre-retail with (partly) no heating during the consumer phase (raw consumption). The exposure per portion was lower for steak tartare than for filet americain because steak tartare was more often frozen during storage and sometimes heated during food preparation. Together this more than compensated the higher portion size of steak tartare.

Finally, veal roulade (rank 6) was delimited by heating profile 6 (Table 6) and product type I. This product was characterized by no EEC reduction during pre-retail and transmission via the rare and medium prepared product and via cross-contamination.

Considering separate food products at population level at the moment of consumption, the exposure to EEC for the Dutch population in a year was highest for filet americain (Table 8, last column), which accounted for 61% (7.16E8/1.17E9) of total EEC exposure through meat consumption and 79% (7.16E8/9.05E8) of total EEC exposure through beef consumption (see Table 7). Chicken fillet and teewurst caused 70% and 30% of total exposure via chicken and pork consumption, respectively. Together the ten products responsible for the highest total exposure accounted for 94% of total exposure through meat consumption. These ten products contained five products that have a relatively low exposure per portion (Table 8, fifth column) but were consumed relatively frequently (Table 8, sixth column): chicken fillet, hamburger, salami, cervelat and chicken drumstick.

**Fig 2:**
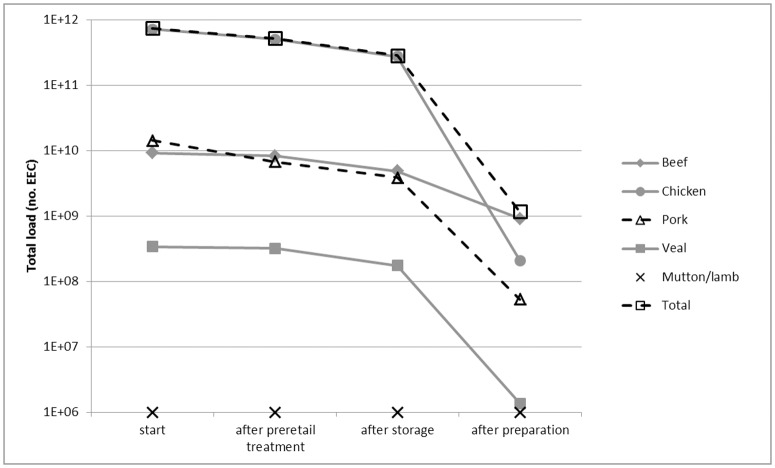
Food animal—specific EEC load. Shown is the load at the start (before any treatment), at retail (after possible preretail treatment), after storage at the consumer and after preparation at the consumer (= at the moment of consumption). The loads refer to the total meat consumption by the whole Dutch population in a year. All Mutton/lamb values (marker X) are zero and inserted arbitrarily at the Y-axis 1E+06 value.

Fig 2 shows the dynamics over the food chain phases of food animal specific EEC load. The load at the start was equal to EEC concentration (CFU/g) x portion size (g) x number of portions (consumed by Dutch population in a year) x EEC prevalence. At the start and after retail and storage, EEC load on chicken meat was highest, being responsible for 97% of total EEC load. EEC load on beef and pork shifted in ranking due to pre-retail processing, which was applied to 10% and 54% of the start load, respectively. After preparation by the consumer, chicken meat dropped to 18% of total EEC load, while the contribution of beef increased to 78% of total load. The relatively large drop in chicken meat EEC load was due to the fact that virtually all EEC on the product were inactivated by heating; the remaining EEC after preparation were almost entirely due to cross-contamination. For the other animals, the drop in EEC load on meat after preparation by the consumer was less than for chicken as for the other animals EEC transmission via the product route was significant, related to the top products of Table 8 (e.g. filet americain, teewurst).

**Fig 3:**
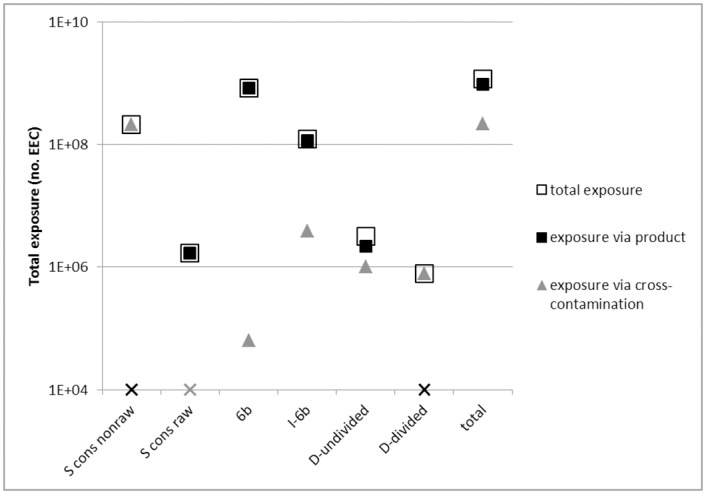
Total exposure and exposure split up into transmission via the meat product and cross-contamination for several product categories. ‘S cons non raw’ and ‘S cons raw’ stand for Surface-contaminated products (see 2.1) that were either heated or not heated by the consumer, respectively. ‘6b’ and ‘I-6b’ are category 6b products and I-products without category 6b, respectively (see Table 2). ‘D-undivided’ and ‘D-divided’ refer to the fraction of Dividable products that were undivided and divided by the consumer during heating, respectively. A marker is changed to an X while retaining black or grey when the value is zero and is inserted arbitrarily at the Y-axis 1E+04 value.

Fig 3 shows the importance of transmission at the consumer phase via the product itself and via cross-contamination, broken down into relevant product categories. This shows that for S-products, which were heated by the consumer (e.g. chicken fillet), and D-products (minced meat), which were divided and heated, all EEC on the product surface were inactivated and all exposure occurred via cross-contamination. Cross-contamination was further only relevant in the category ‘D-undivided’ (meat ball). In the categories 6b (e.g. filet americain) and I without 6b (e.g. steak tartare) cross-contamination was minor and in S-products that were not heated by the consumer (e.g. smoked beef) it was absent. Overall, (2.15E8/1.17E9)*100% = 18% of exposure was transmitted via cross-contamination.

**Fig 4:**
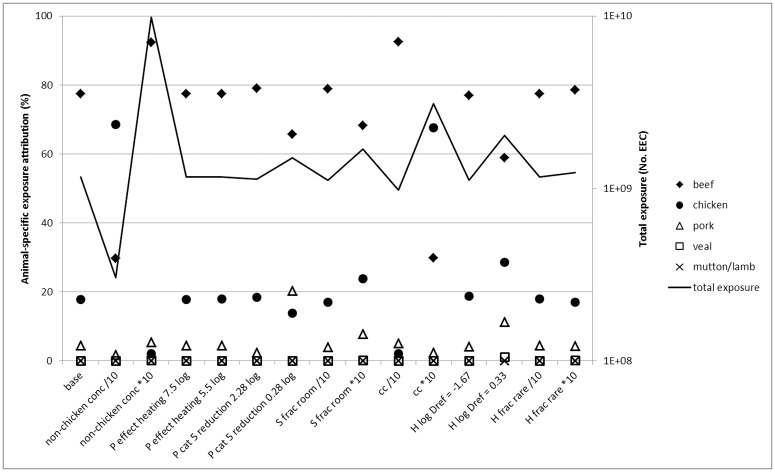
Sensitivity analysis results. Effect of 10-fold changes in parameter base values on total exposure (No. EEC) and food animal-specific attribution of exposure. P = preretail, S = storage, H = consumer heating, conc = concentration, cat = category, frac = fraction, cc = cross-contamination.

Sensitivity analysis showed that the effect of parameter values on model output in terms of total exposure was generally limited (Fig 4). However, a large effect was found for EEC concentrations for meat from other animals than chicken (non-chicken conc; overall exposure 3.8 times lower and 8.4 times higher than base scenario) and to a lesser extent the fraction of EEC transmitted by cross-contamination (cc). For the relative contribution of meat originating from different animals species (Fig 4), the same parameters gave the largest effect: for both a 10-fold lower EEC concentration for meat from other animals than chicken (non-chicken conc/10) and 10-fold higher cross-contamination (cc*10), the exposure attribution shifted from 18% chicken and 78% beef (base scenario) to 68% chicken and 30% beef, respectively. In the reverse situation, it became 2% chicken and 92% beef, and the contribution of pork to the total exposure through meat consumption became higher than that of chicken meat. Less important shifts were found for one log_10_ less pre-retail reduction for category 5 (P cat 5 reduction 0.28 log; dry sausages): this caused the contribution for pork to rise to 20%, higher than for chicken. A one log_10_ higher value for *D*_*ref*_ (H log Dref = 0.33; decimal reduction time for heating, see 2.1) lead to a relatively large increase in the contribution for pork and chicken and a decrease for beef.

## 4. Discussion

In this study, the total exposure of humans to EEC in The Netherlands per year through the consumption of meat was quantified. In addition, the contribution of meat and specific meat products originating from different food animals to the total exposure through meat consumption was estimated. An unexpected finding of the present study was that beef- and not chicken meat resulted in the highest exposure to EEC, despite the very high prevalence of EEC in broilers [1] and on raw chicken meat at retail [10]. The reason was that certain beef products were consumed raw (filet americain and ossenworst) and that the preparation process by the consumer (heating and cross-contamination) gave a larger decrease in EEC load for chicken products compared to products from other animals. A Belgian study assessing human exposure to 3^rd^ generation cephalosporin-resistant *E*. *coli* found that the probability to be exposed to 10 CFU or more by the consumption of chicken meat was 7.0% [50]. This was somewhat higher than the present model output: a probability to be exposed to 1 CFU or more by the consumption of chicken meat of 6.85% with a mean exposure of 1.75 CFU per contaminated portion (Table 7). Depoorter *et al*. [50] concluded that the majority of exposure through chicken meat was caused by cross-contamination, which was in agreement with the present findings.

The sensitivity analysis gave reasonable confidence in the model results (Fig 4). For specific meat products, however, the effect of parameter value change on EEC exposure could be large. EEC concentrations for meat from other animals than chicken and cross-contamination were the parameters with the largest effect on model output. EEC concentrations for meat from other animals than chicken was a source of uncertainty as it was based on *E*. *coli* concentrations and on the assumption of a fixed EEC / *E*. *coli* ratio. Future research should investigate EEC concentrations on meat from other food animals than chicken. The cross-contamination parameter value was less uncertain, being based on the results of a literature review (see 2.2.6).

The objective of the Dutch National Food Consumption Surveys was to establish data on food and nutrient intake and was not created for the microbiological working field. The design used two 24h time frames, so that the consumption data for rarely consumed but possibly important products were relatively uncertain (e.g. teewurst, Table 8). Important data on storage and heating were not considered. Chardon and Swart [31] partly filled these data gaps, but more extensive data are needed.

It was assumed that food was heated according to the recommendations in a cookbook [37]. The undoubtedly occurring deviations from the ‘ideal cook’ are expected to affect absolute exposure estimates more than attribution estimates. Further, conflicting results were found in the literature on the inactivating effect of heating (*D*_*ref*_), see e.g. de Jong *et al*. [76].

Model extensions in the future are possible and desirable, related to the very large diversity of meat products and ways of consumer storage and preparation. Suggestions are listed below.

- include minor food categories, such as turkey, game, horse, giblets and snacks (e.g. croquette).
- include the potentially important raw beef product carpaccio (limited consumption data).
- include the possible effect of food processors injecting meat with fluids or tenderizing meat.
- include the possible inactivating effect of marinating.
- differentiate in the effect of heating (now a 6.5 log_10_ reduction) for the different pre-retail categories.
- investigate in more detail the issues of pre-retail heating of chicken products and of schnitzels and other meat products consisting of aggregated small pieces of meat (i.e. non-intact meat).
- include the effect of pre-retail freezing of meat, be it during transport of imported meat, or during storage at the butcher/slaughter house.
- Lahou *et al*. [75] showed that meat temperature will initially remain high on the serving plate. Include this additional inactivating effect.
- include possible reheating of food (e.g. microwave).
- include variability and uncertainty in EEC concentrations and parameter values when more data become available. This was not done in the present model as this would ask for a great number of assumptions on probability distributions and parameter values not leading to firmer conclusions.

To date it is unknown if exposure to EEC through the consumption of meat leads to carriage of EEC and whether this eventually results in a significant health burden in humans. It should be noted that the estimated number of EEC ingested per portion was low. The mean number of EEC on a contaminated portion of meat at the moment of consumption was estimated to be 6.15, which combined with the fraction of contaminated portions of 0.0155 equalled the mean number of 0.0955 EEC per portion of meat (Table 7). As yet, a modelling framework to calculate the public health risk of antimicrobial resistance is not available. The standard QMRA chain models for pathogenic microorganisms need to be adjusted in order to model EEC carrier prevalence and health burden. The exposure model used here would have to be extended with a dose-response relationship for EEC-carriership and in addition would have to include:

- all relevant food, environment and direct animal-to-human contact transmission routes;
- bi-directionality of transmission routes;
- human-to-human transmission;
- transmission-route specific EEC dose response;
- the rate at which human carriers lose their EEC;
- horizontal transmission of ESBL plasmids and/or genes.

Dynamic models (e.g. compartmental models based on ordinary differential equations) explicitly capture time and can therefore account for bi-directional pathways. They might prove an alternative.

Next, EEC carriership must be linked to the health burden of relevant diseases, such as urinary tract infections (UTIs) [3], by a mathematical model. The EEC-related health burden (e.g. expressed in Disability Adjusted Life Years (DALYs)) for a specific disease equals the increase in health burden in the present situation (i.e., with a given EEC prevalence) compared to a scenario without EEC carriers. This increase can be related to a higher incidence of the specific disease, a higher DALY/case value, or both.

Research into the following other reservoirs or transmission routes for EEC has been done: retail vegetables [77], swimming in recreational water [78], direct contact with broilers [1] and human-to-human transmission [79]. This did not lead to human EEC exposure estimates that could be compared with exposure via meat consumption, because quantitative data were scarce [4] and the intensity of human exposure was seldom investigated.

For the individual consumer, the EEC exposure per portion (= EEC exposure per contaminated portion x EEC prevalence) is the determinative exposure variable. From this perspective, individual risk management would imply avoiding raw meat products as these clearly resulted in the highest exposure per portion (Table 8). For the authorities, exposure at population level (per food animal or specific meat product) is more important: reduction of this exposure can potentially reduce the public health risk. Possible interventions could include:

- Farm: reduction of the EEC prevalence;
- Slaughter house: improvement of hygiene;
- Distribution and Retail: reduction of temperature abuse and cross-contamination;
- Consumer:
  - reduction of consumption of products with a high exposure per portion and at population level.
  - reduction of cross-contamination (covers 18% of transmission).

One way of reducing the EEC prevalence in food animals is to decrease the amount of antimicrobials used in veterinary medicine. Between 2009 and 2014 the total sales of antibiotics in veterinary medicine decreased by 58%. In 2014, resistance rates of *E*. *coli* isolated from poultry meat have markedly decreased compared to 2013 probably due to the decrease in antibiotic usage in poultry in the Netherlands. In 2014 the EEC prevalence in poultry meat was 67% (the parameter used in the present model), which was lower than found in 2013 (83%) and in 2012 (73%) [10]. A possible further change in prevalences of resistance in livestock due to alterations in consumption patterns of antimicrobials will affect model output.

The main part of exposure was due to 1) raw beef products (filet americain, ossenworst) via the product itself and 2) chicken fillet via cross-contamination (Table 8, Figs 2 and 3). Options to influence the consumer include television/internet commercials, primary/secondary school education and warning labels on meat products. In the Netherlands, chicken fillet is sold with a warning label that stresses the danger of cross-contamination and undercooking in relation to bacteria. A label that warns for the microbiological risk of raw beef products (which includes STEC O157 [80]) could be considered, next to the less realistic option of forbidding these products.

In conclusion, consumption of beef products led to a higher EEC exposure than chicken products, where filet americain and chicken fillet were the specific products with the highest exposure. Pork and veal products accounted for only 4.5% and 0.1% of the total EEC exposure, although pork was consumed most frequently compared to meat from other animals. Future work will focus on investigating and modelling the relationship between EEC exposure and public health risk.
