# Correction: Comparative Exposure Assessment of ESBL-Producing *Escherichia coli* through Meat Consumption

**DOI:** 10.1371/journal.pone.0173134

**Published:** 2017-02-24

**Authors:** The PLOS ONE Staff

Fig 1 appears incorrectly in the published article. Please see the correct Fig 1 and its caption here. The publisher apologizes for the error.

**Fig 1:**
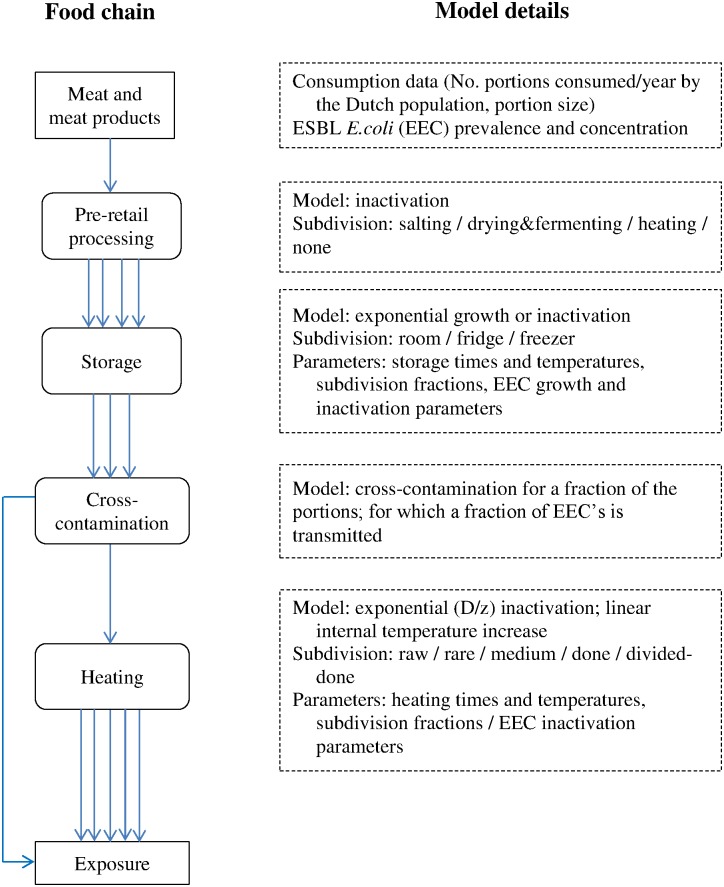
Overview of the sQMRA model used for the calculations. In- and output (text box with straight corners), processes (rounded corners) and details (dashed lines) are shown.
